# Prioritizing conservation in sub‐Saharan African lakes based on freshwater biodiversity and algal bloom metrics

**DOI:** 10.1111/cobi.13914

**Published:** 2022-05-26

**Authors:** Cody Danaher, Tim Newbold, Jeffrey Cardille, Abbie S. A. Chapman

**Affiliations:** ^1^ Centre for Biodiversity and Environment Research, Department of Genetics, Evolution and Environment University College London London UK; ^2^ Department of Natural Resource Sciences and Bieler School of Environment McGill University Montreal Québec Canada; ^3^ UCL Institute for Sustainable Resources London UK

**Keywords:** algal blooms, freshwater biodiversity, Google Earth Engine, lakes, remote sensing, species richness, sub‐Saharan Africa, África sub‐sahariana, biodiversidad de aguas dulces, floración de algas, Google Earth, lagos, riqueza de especies, teledetección, 藻华, 谷歌地球引擎, 淡水生物多样性, 湖泊, 遥感, 物种丰富度, 撒哈拉以南非洲地区

## Abstract

As agricultural land use and climate change continue to pose increasing threats to biodiversity in sub‐Saharan Africa, efforts are being made to identify areas where trade‐offs between future agricultural development and terrestrial biodiversity conservation are expected to be greatest. However, little research so far has focused on freshwater biodiversity conservation in the context of agricultural development in sub‐Saharan Africa. We aimed to identify lakes and lake areas where freshwater biodiversity is most likely to be affected by eutrophication and Harmful Algal Blooms (i.e., when algae multiply to the extent that they have toxic effects on people and freshwater fauna), some of the most important emerging threats to freshwater ecosystems worldwide, especially with the onset of climate change. Using novel remote‐sensing techniques, we identified lakes that demonstrated high biodiversity and algal bloom levels. We calculated the richness of freshwater species and the normalized difference chlorophyll index (NDCI) to prioritize lakes in Ghana, Ethiopia, Zambia, and bordering countries, of high priority for conservation. We identified 169 priority lakes and lake areas for conservation, based on high levels of biodiversity exposed to potentially harmful algal blooms. Zambia had the most lakes identified as conservation priorities (76% of its small lakes and five 100‐km^2^ areas in large lakes). Many of the conservation priority lakes and lake areas identified in this study were in transboundary watersheds; thus, collaborative water resource management and conservation at the watershed scale is needed. The use of remote‐sensing tools to prioritize freshwater systems for conservation according to algal‐bloom risk is vital in remote, undersampled world regions, especially given the increasing threat posed to freshwater biodiversity by rapidly expanding agriculture and climate change.

## Abstract (es)

Priorización de la Conservación en los Lagos Sub‐Saharianos con base en Medidas de Biodiversidad de Aguas Dulces y Floración de Algas

**Resumen:**

Conforme el cambio climático y el uso de suelo para cultivos siguen representando amenazas crecientes para la biodiversidad en la región sub‐sahariana de África, se están realizando esfuerzos para identificar las áreas en donde se espera que sucedan las mayores compensaciones entre el desarrollo agrícola venidero y la conservación de la biodiversidad terrestre. Sin embargo, pocas investigaciones se han centrado en la conservación de la biodiversidad de aguas dulces dentro del contexto del desarrollo agrícola en esta región de África. Nos enfocamos en localizar las áreas en donde sea más probable que la biodiversidad de aguas dulces se vea afectada por la eutrofización y las floraciones de algas (es decir, cuando las algas se multiplican a tal grado que tienen efectos tóxicos sobre las personas y la fauna de agua dulce), dos de las amenazas emergentes más importantes para los ecosistemas de agua dulce en todo el mundo debido al cambio climático. Mediante técnicas novedosas de teledetección identificamos los lagos que se traslapaban con áreas de gran biodiversidad y floraciones de algas. Calculamos la riqueza de especies de agua dulce y el índice de diferencia normalizada de clorofila (IDNC) para identificar los lagos de suma importancia para la conservación en Ghana, Etiopía, Zambia y sus países fronterizos. Identificamos 169 áreas prioritarias para la conservación con base en los niveles elevados de biodiversidad expuestos a las floraciones de algas potencialmente dañinas. Zambia tuvo la mayor cantidad de lagos identificados como prioridades de conservación (76% de sus lagos pequeños y cinco áreas de 100 km^2^ en los grandes lagos). Las amenazas para la biodiversidad de agua dulce estuvieron presentes a nivel de cuenca, con frecuencia con una extensión más allá de las fronteras políticas de un país; por lo tanto, se requiere que el manejo de recursos hídricos y la conservación sean esfuerzos colaborativos a nivel de cuenca. El uso de herramientas de teledetección para priorizar la conservación de los sistemas de agua dulce de acuerdo con el riesgo de floración de algas es vital en las regiones remotas y poco muestreadas del mundo, especialmente debido a la amenaza creciente que representan el cambio climático y la expansión agrícola para la biodiversidad de agua dulce.

## 根据淡水生物多样性和藻华指标确定撒哈拉以南非洲湖泊的优先保护地区

**【摘要】** 由于农业用地和气候变化持续对撒哈拉以南非洲地区的生物多样性构成严重威胁, 人们目前正在努力确定未来农业发展和陆地生物多样性保护之间预计有最优利弊权衡的地区。然而, 到目前为止, 很少有研究关注撒哈拉以南非洲地区农业发展背景下的淡水生物多样性保护问题。本研究旨在确定淡水生物多样性最有可能受到富营养化和有害藻华爆发影响(即当藻类繁殖到对人和淡水动物产生毒性影响的程度)的地区, 这些因素是随着气候变化出现的对全球淡水生态系统最重要的威胁。我们利用新型遥感技术, 确定了与高生物多样性和藻华爆发的地区相重叠的湖泊, 并计算了淡水生物物种丰富度和归一化叶绿素指数(NDCI), 以确定加纳、埃塞俄比亚、赞比亚和周边国家应高度优先保护的湖泊。我们根据高生物多样性在有害藻华爆发中的暴露情况, 确定了169个优先保护区域。其中, 赞比亚应优先保护的湖泊数量最多(76%的小湖泊以及大湖泊中5个100平方公里区域)。淡水生物多样性的威胁因素存在于流域尺度, 往往超出了一个国家的政治边界;因此, 需要在流域尺度上进行水资源的合作管理和保护。利用遥感工具来根据藻华爆发风险确定淡水系统的优先保护区域, 对于全球采样不足的偏远地区至关重要, 特别是考虑到快速扩张的农业和气候变化正在对淡水生物多样性的威胁正在日益加剧。**【翻译:胡怡思;审校:聂永刚】**

## INTRODUCTION

As the global human population and associated food demand continue to increase, so does the need to use land for agriculture (Tscharntke et al., 2012). A large proportion of the world's potential agricultural land lies in sub‐Saharan Africa, making this region a major focus for global agricultural development in the coming decades (Delzeit et al., 2017; Roxburgh et al., 2010; van Ittersum et al., 2016). One of the greatest environmental trade‐offs associated with agricultural development in sub‐Saharan Africa is likely to be a reduction of biodiversity and ecosystem services (FAO, 2016; Perrings & Halkos, 2015). Globally, agricultural expansion is a leading cause of biodiversity loss via habitat fragmentation, homogenization and removal (e.g., Newbold, 2018; Newbold et al., 2019). Climate change is another key driver of biodiversity loss. Its effects are being felt at multiple scales, from individuals to whole ecosystems (Bellard et al., 2012). Biodiversity losses compromise the resilience of ecosystems to environmental and anthropogenic stressors, impair their functioning , and reduce the provision of ecosystem services, to the detriment of humans (e.g., Cardinale et al., 2012; Oliver, Isaac, et al., 2015; Oliver, Heard, et al., 2015). Given that most unfarmed land in sub‐Saharan Africa is covered by natural habitats, such as forests and savannahs (Estes et al., 2016; FAO, 2016; Kehoe et al., 2017), and that rural livelihoods depend heavily on the ecosystem services supported by biodiversity (Bourne et al., 2016; Roe, 2010), addressing this biodiversity–agriculture trade‐off is urgent, as is the need to prepare for the consequences of climate change.

Some of the most important ecosystem services supporting livelihoods are derived from freshwater ecosystems, such as rivers, lakes, and wetlands (e.g., Kafumbata et al., 2014; McClain, 2013; Sayer, Máiz‐Tomé, et al., 2018). From providing food and water to protecting communities from extreme flooding events or droughts, freshwater ecosystems are crucial to human existence, transporting nutrients and providing health and recreational benefits (Albert et al., 2021; Dudgeon et al., 2006; Vörösmarty et al., 2010). Freshwater ecosystems are also crucial for biodiversity conservation (e.g., Collen et al., 2014). Despite covering only a small portion of Earth's surface, freshwater ecosystems harbor 6.0–9.5% of all described species (Dudgeon et al., 2006; Reid et al., 2019). Freshwater biodiversity is also at very high risk globally (Vörösmarty et al., 2010) and may be declining at a much faster rate than terrestrial biodiversity (Darwall et al., 2011; Dudgeon et al., 2006; Reid et al., 2019; but see van Klink et al., 2020). Despite its importance, freshwater biodiversity is relatively undersampled and understudied (Abell, 2002; Holland et al., 2012; McManamay et al., 2018). In sub‐Saharan Africa, freshwater biodiversity has only begun to be investigated systematically in the last few decades (e.g., Collen et al., 2014; Darwall et al., 2011; Holland et al., 2012;).

One of the most important mechanisms through which agricultural development threatens freshwater ecosystems is through excessive runoff of nutrients, which drives eutrophication, the process through which water bodies accumulate nutrients, such as nitrogen and phosphorous. In eutrophic aquatic systems, limits to optimal plant and algal growth are exceeded, and, ultimately, dissolved oxygen is depleted to fatal levels (Ansari et al., 2010; Attua et al., 2014; Smith, 2003). The eutrophication of water bodies can also be caused by rapid inputs of organic matter from natural sources, such as flash floods or extreme weather events, the frequency of which is likely to increase with climate change (e.g., Nazari‐Sharabian et al., 2018; Schindler et al., 2012). Climate change and agricultural development may, therefore, pose a synergistic threat to freshwater biodiversity via eutrophication.

Eutrophication, in turn, increases the probability of Harmful Algal Blooms (HABs). In an HAB event, cyanobacteria (blue‐green algae that produce toxins that decrease water quality for freshwater organisms and the humans and animals that drink the water) represent more than half of the algal biomass in a waterbody (Ansari et al., 2010; Smith, 2003; van Soesbergen et al., 2019). These cyanobacteria‐dominated blooms are one of the most important emerging threats to freshwater ecosystems worldwide (Reid et al., 2019) and are expected to increase in duration and frequency as the climate changes due to competition effects between cyanobacteria and other algae made possible at higher temperatures (Havens & Paerl, 2015; O'Neil et al., 2012; Paerl & Huisman, 2009). HABs have been observed in lakes and reservoirs in sub‐Saharan Africa (Addico et al., 2017; Dejene, 2009; Ndlela et al., 2016), but the extent to which they possibly coincide with areas rich in freshwater biodiversity has yet to be established.

Remote‐sensing techniques offer a means to understand and map algal blooms (e.g., Bresciani et al., 2018; Caballero et al., 2020). The most suitable satellites for this are the SENTINEL 2A and 2B satellites, launched in 2015 and 2017, respectively (e.g., Beck et al., 2016, 2017; Rodríguez‐Benito et al., 2020). These satellites have a fine spatial and temporal resolution. They take images of the same location on Earth every 5 days at a band‐dependent spatial resolution of 10–60 m. This, and their 12‐bit radiometric resolution, enables these satellites to capture low‐reflectance objects of relatively small size, such as lakes (Bresciani et al., 2018; Shi & Wang, 2014; Toming et al., 2016). Data from these satellites are freely accessible via Google Earth Engine (Gorelick et al., 2017). Nevertheless, the use of remote‐sensing tools for freshwater systems in sub‐Saharan Africa has so far mostly been limited to assessing the water quality of the African Great Lakes (Dube et al., 2015). To our knowledge, no one has used remotely sensed data to assess overlap between high richness of freshwater species and high risk of HABs in smaller African inland waters, despite the importance of this for the conservation of freshwater biodiversity.

We aimed to identify priority lakes and lake areas for freshwater biodiversity conservation based on the overlap between high freshwater biodiversity and algal blooms in 3 countries that span different agroecological zones of sub‐Saharan Africa: Ghana, Ethiopia, and Zambia (Appendix S0) (countries central to the Sentinel project [Sentinel, 2018]).

## METHODS

### Study area and spatial scale

**FIGURE 1:**
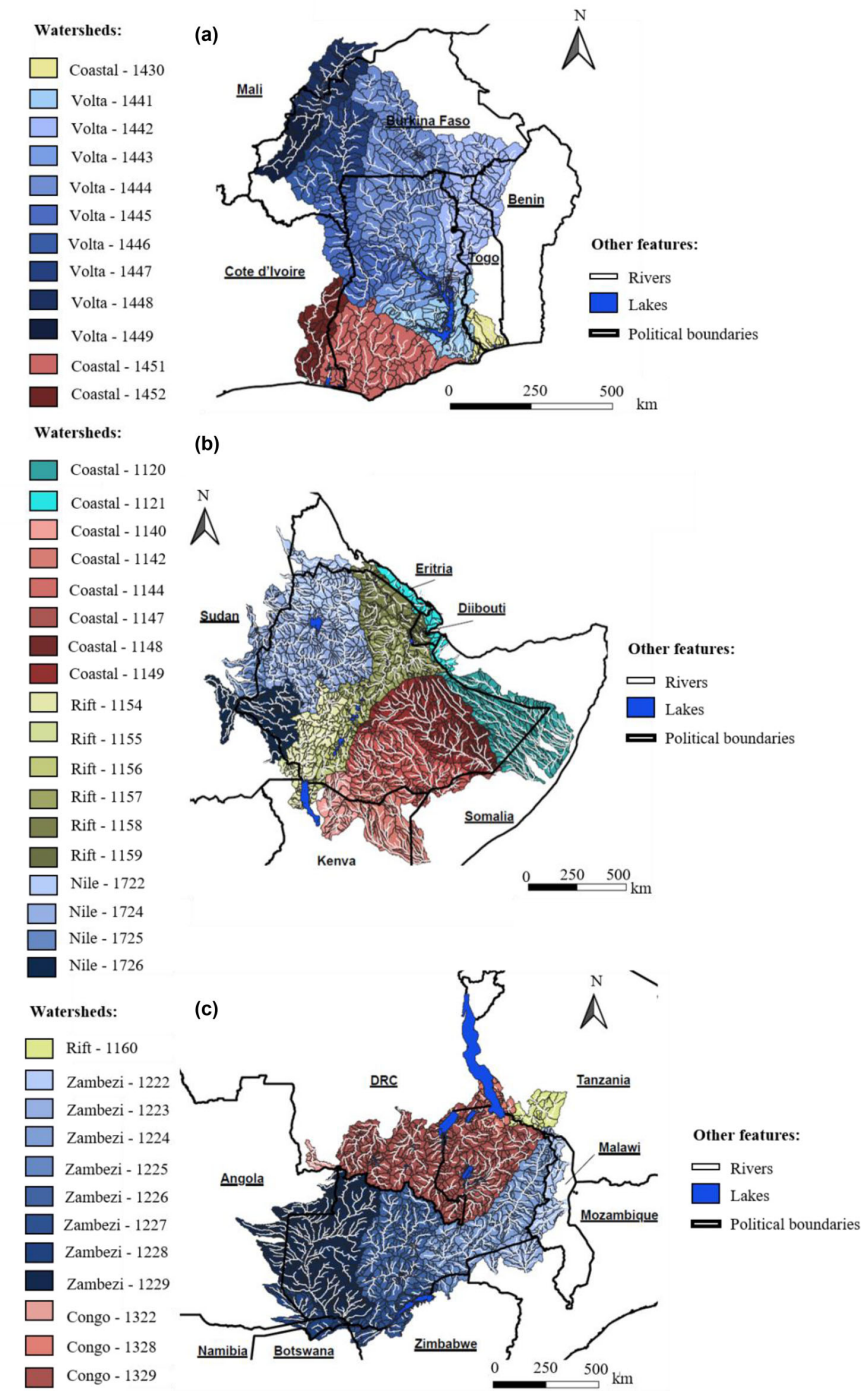
Hydrologically relevant watersheds in the study area: (a) Ghana, (b) Ethiopia, and (c) Zambia. Watershed colors are based on level 3 and level 4 HydroBASINS (see Lehner & Grill, 2013)

We used the freely available HydroBASINS dataset (Lehner & Grill, 2013) and QGIS (QGIS Development Team, 2019) to identify watersheds that were partially or wholly within the political borders of Ghana, Ethiopia, and Zambia (defined using the Global Administrative Areas database [Global Administrative Areas, 2012]) (Figure 1; Appendix S0). It is recommended that water resources, and associated ecosystems, be managed at the watershed scale (Nguyen et al., 2016). Furthermore, although the eutrophication of lakes is highly dependent on land‐use patterns, it is also shaped by lake and watershed characteristics (e.g., Khan & Mohammad, 2013; Kim et al., 2016; Soranno et al., 1996).

### Measuring freshwater biodiversity

For both obligate freshwater species and freshwater‐dependent vertebrate species in each country, we used the HydroBASINS data set (format 2 [i.e., inserted lakes]) and International Union for the Conservation of Nature (IUCN) datasets. The HydroBASINS dataset provides nested watershed boundaries across the globe and is used by the IUCN to reference the location of some freshwater species (Lehner & Grill, 2013; McManamay et al., 2018). Therefore, we combined IUCN freshwater HydroBASINS tables, which link fish, crab, crayfish, mollusc, odonate, and shrimp species to specific river or lake catchment units in the HydroBASINS dataset (Lehner & Grill, 2013), to calculate the number of obligate freshwater species per HydroBASIN.

We identified for which species of mammals, birds, amphibians, and reptiles freshwater habitats (i.e., wetlands, rivers, lakes, ponds, and streams) are of “major importance” to the survival of the species according to the IUCN Red List database (IUCN, 2019). A habitat is considered of major importance to a species’ survival when it is essential for a particular stage of the species’ life cycle or when it is the primary habitat of the species (IUCN, 2022). These species were considered freshwater‐dependent vertebrate species. We intersected the IUCN extent‐of‐occurrence distribution polygons for these species with the HydroBASINS (i.e., watersheds) included in our analyses with the sf package in R (R Core Team, 2019; Pebesma, 2018). We assumed species were present in all locations in the published extent‐of‐occurrence distribution maps, which is likely to lead to overestimation of biodiversity in some areas (Herkt et al., 2017), but should be sufficient for establishing broad‐scale patterns of biodiversity. The dataset derived from these intersections comprised species names, IUCN Red List categories, and watershed identifiers matching the HydroBASINS (i.e., watersheds) with which a given species range intersected. We used this new dataset to compute the number of freshwater‐dependent vertebrate species per watershed.

Finally, we mapped: obligate freshwater species per watershed; obligate freshwater species per IUCN Red List category per watershed; freshwater‐dependent vertebrate species per watershed; freshwater‐dependent vertebrate species per IUCN Red List category per watershed; a combined species count (freshwater and freshwater‐dependent vertebrate species) per watershed; and a combined species count per IUCN Red List category per watershed with QGIS (QGIS Development Team, 2019).

### Normalized difference chlorophyll index

We used Google Earth Engine (Gorelick et al., 2017) to compute the normalized difference chlorophyll index (NDCI) (see Equation 1). We used the NDCI values as indicators of algal content (Mishra & Mishra, 2012; Kamerosky et al., 2015) in Sentinel‐2 satellite imagery for each of the 1812 lakes in our study areas in the HydroLAKES database. The HydroLAKES dataset defines the lakeshore boundaries of all global lakes with an area >0.1 km^2^ (Messager et al., 2016). Sentinel‐2 is well‐suited for monitoring algal blooms in both large and small lakes (Beck et al., 2016; Caballero et al., 2020). We accessed and computed the NDCI for all scenes with <10% cloud cover (*n* = 26,560 scenes; Ghana, 4998 scenes; Ethiopia, 6856; Zambia, 14,706) from January 1 to December 31 of each year from 2016 to 2018 for further processing. We set a 10% cutoff value for observations; it indicated presence of clouds in the short wave infrared band, which is near 0 over water in cloudless imagery (Shi & Wang, 2014). For band 11, cutoff values were 2990 for Ghana, 1680 for Ethiopia, and 1510 for Zambia. A small number (*n* = 35) of lakes did not have clear imagery in this time span; these were removed from further consideration. Lakes >100 km^2^ (*n* = 27) were divided into multiple 100‐km^2^ sections to detect potential blooms that did not cover their full surface area. We computed the mean, median, and standard deviation of NDCI values for the water pixels of each lake or lake section for each date. Then, the median NDCI statistic calculated from each cloud‐free observation for each lake was temporally averaged over the 3 years (i.e., 2016–2018). This produced a single NDCI value for each of the lake features across all 3 years. This value was retained for conservation prioritization of lakes. The median NDCI statistic added to each satellite observation of each lake was considered the most accurate estimate of NDCI across the surface of each lake because, if potential inaccuracies existed in any lake's geographic boundaries, land reflectance biases would have affected the mean NDCI more than the median NDCI given the nature of these 2 statistics:

(1) $$\mathrm{NDCI}=\frac{\rho \mathrm{NIR}\left(708\;\mathrm{nm}\right)\;-\;\rho \mathrm{red}\left(665\;\mathrm{nm}\right)}{\rho \mathrm{NIR}\left(708\;\mathrm{nm}\right)\;+\;\rho \mathrm{red}\left(665\;\mathrm{nm}\right)},$$

where ρ is the surface reflectance for the respective spectral band, red are the red bands, and NIR are the red near‐infrared bands (Mishra & Mishra, 2012).

### Prioritizing lakes for conservation

We prioritized lakes for conservation under the assumption that high levels of eutrophication and sustained algal blooms have a negative effect on freshwater communities (Chorus & Bartram, 1999; Landsberg, 2002; O'Neil et al., 2012). To be considered a lake (or lake area, for large lakes) of high conservation priority, 2 criteria had to be met: temporally averaged median NDCI > 0.1 and freshwater species richness above the median richness for the country. These criteria were applied either to whole lakes, if their area was <100 km^2^, or to portions (100 km^2^ grid squares) of larger lakes. Areas where NCDI was ≥0.1 (chlorophyll concentration ≥25 mg/m^3^) were considered at risk from HABs (see “algal bloom flag” thresholds in Binding et al. [2018], who suggest chlorophyll concentrations of >10 mg/m^3^ as a threshold, and NDCI equivalency details in Mishra & Mishra [2012]) (Table 2). We were more conservative in our choice of threshold than Binding et al. (2018) because if a lake has a temporally averaged median NDCI value ≥0.1, a significant portion of the lake must be above this threshold and, thus, be exhibiting high chlorophyll biomass content for an amount of time sufficient to raise the median NDCI value to this level, given that clear water bodies have NDCI values close to −1 (Mishra & Mishra, 2012).

## RESULTS

### Freshwater biodiversity patterns

**FIGURE 2:**
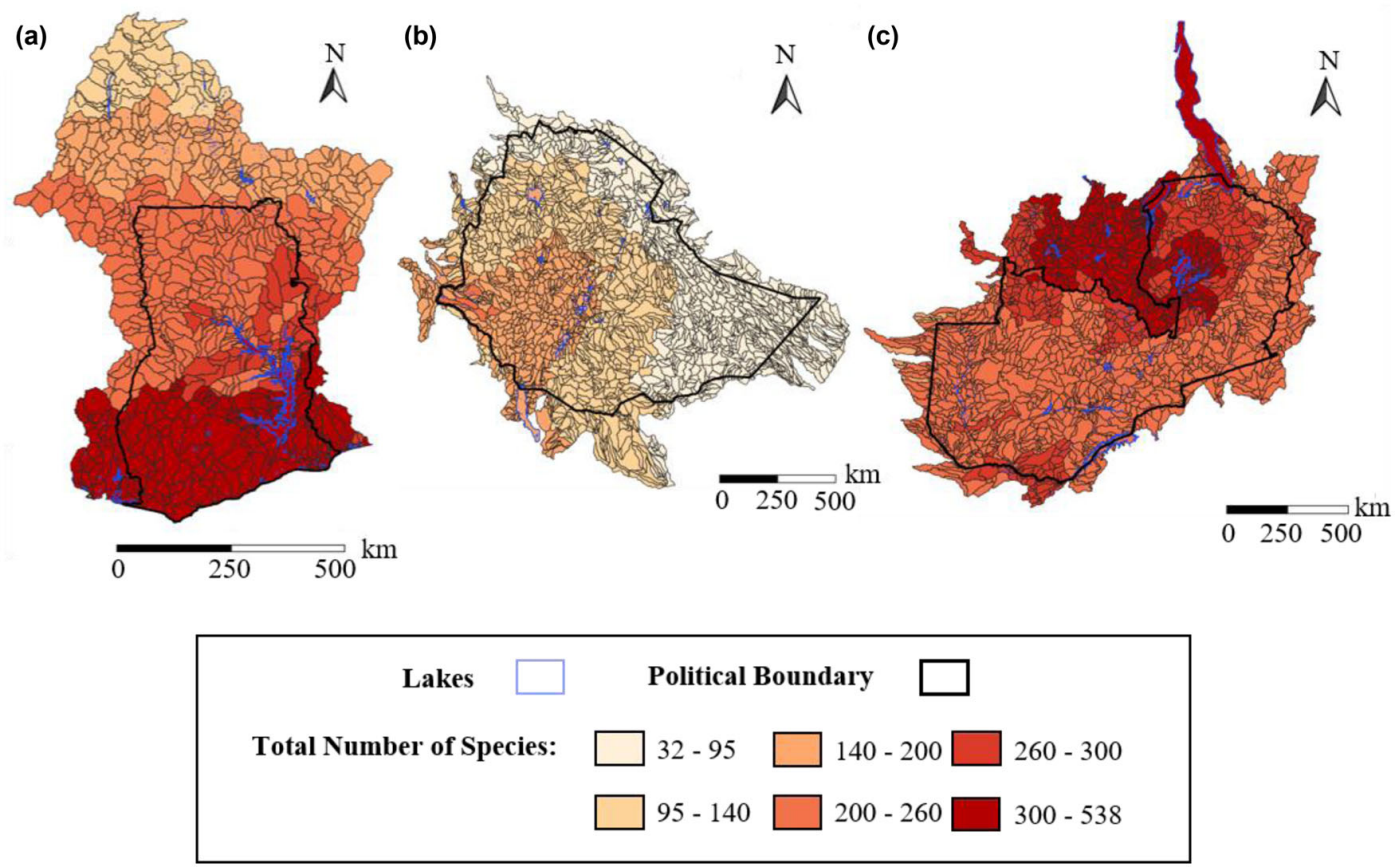
Richness of obligate freshwater species and freshwater‐dependent vertebrate species in (a) Ghana, (b) Ethiopia, and (c) Zambia. Counts of obligate and freshwater‐dependent species in each watershed were combined to produce this richness measure. The watersheds considered stretch beyond country boundaries. Lakes, such as Lake Volta and Tanganyika, are considered watersheds in the customized HydroBASINS dataset and have their own species counts.

**FIGURE 3:**
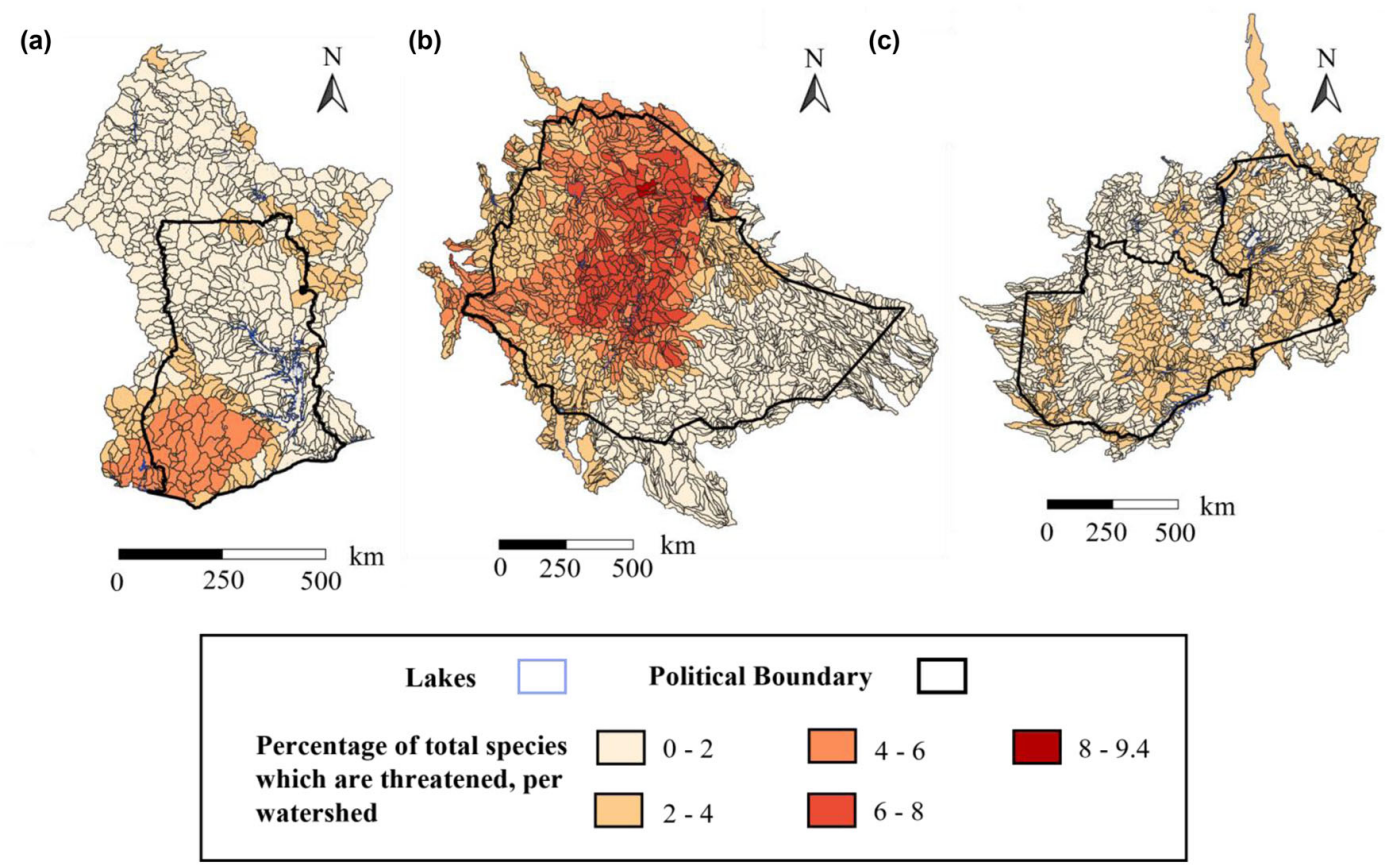
Percentage of species that are classified as threatened (vulnerable, endangered, and critically endangered obligate freshwater species and freshwater‐dependent vertebrate species) per watershed

The total number of freshwater species whose ranges overlapped the watersheds of Ghana, Ethiopia, and Zambia showed strong spatial gradients (total range: 32–538 species per watershed; Ghana, 56–376; Ethiopia, 32–108; Zambia, 81–538) (Table 1a; Figure 2a). Zambia had the highest estimated mean freshwater species richness in its watersheds (mean = 266) (Table 1); only 1% of the watersheds had fewer than 200 species (Figure 2c). Zambia's Lake Tanganyika had the highest potential species richness (*n* = 538). Spatial patterns in freshwater biodiversity were similar for freshwater‐dependent vertebrates (Appendix S1) and obligate freshwater species (Appendix S1), although richness was more concentrated across a smaller area for obligate freshwater species than for freshwater‐dependent vertebrates (Appendix S1). Spatial patterns for threatened species were generally similar to those for all species combined (Figure 3; Appendix S1).

**TABLE 1:** Freshwater species richness estimates across watersheds in Ghana, Ethiopia, and Zambia and normalized difference chlorophyll index (NDCI) values across lakes in each country

	Freshwater species richness						NDCI^a^		
Country	Minimum	Maximum	Range	Mean	Median	Minimum	Maximum	Mean	Median
Ghana	56	376	320	242	237	–0.06	0.21 ^a^	0.01	0.00
Ethiopia	32	208	176	109	106	–0.12	0.23 ^a^	0.00	–0.01
Zambia	81	538	457	266	252	–0.09	0.34 ^a^	0.00	–0.02
All	32	538	506	190	194	–0.12	0.34 ^a^	0.00	–0.02

*Note*: Values that may suggest the area could have potential for present or future harmful algal blooms (≥0.1) are underlined.

^a^ Value shows potential for present or future harmful algal blooms (≥0.1) (see METHODS section).

### Algal blooms

**FIGURE 4:**
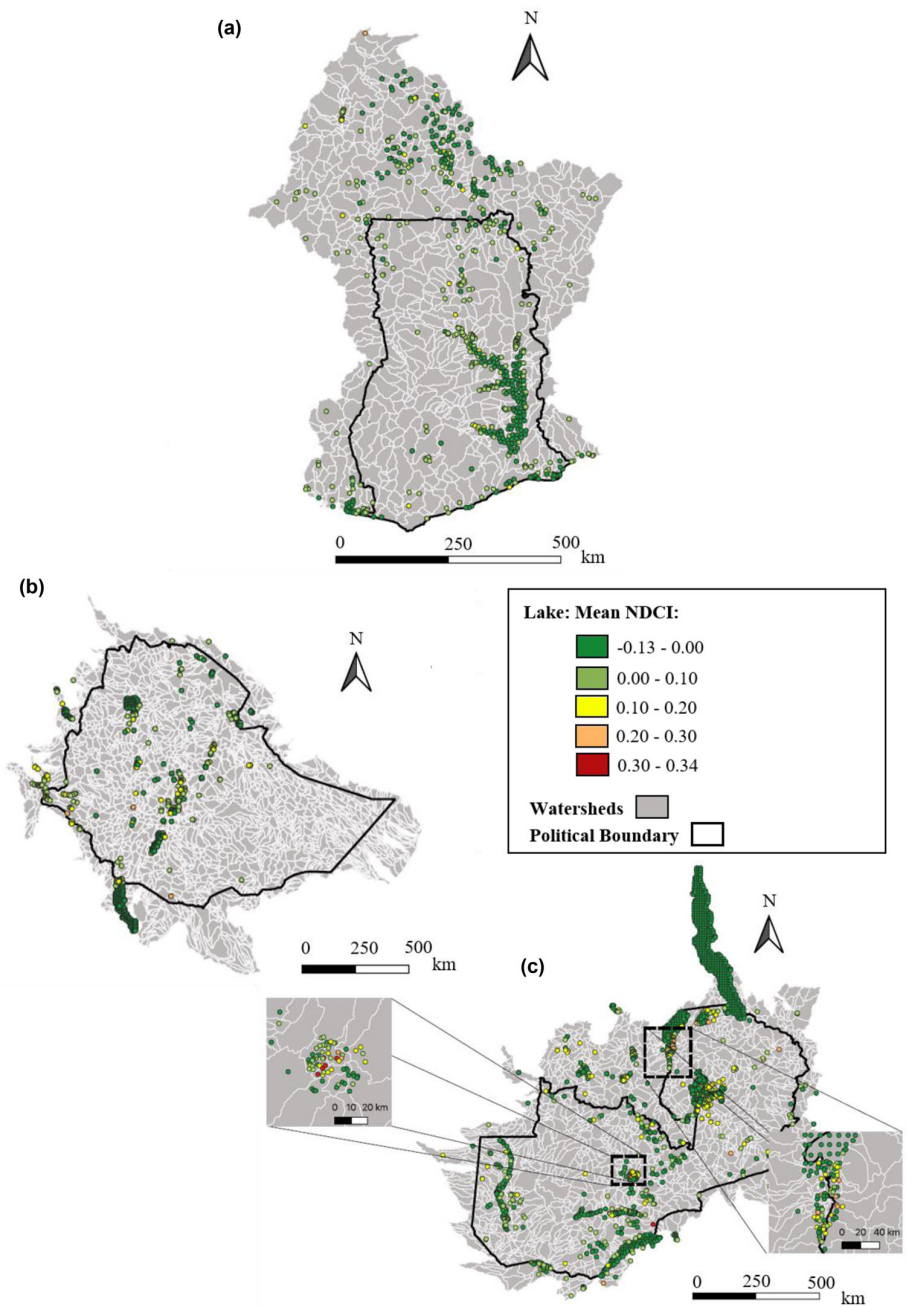
Temporally averaged (2016–2018) median normalized difference chlorophyll index (NDCI) values for all lakes in each of the watersheds that overlap the study countries: (a) Ghana, (b) Ethiopia, and (c) Zambia. Lakes larger than 100 km^2^ in area (e.g., Lake Volta in Ghana) are divided into multiple subsections (see METHODS section).

Algal blooms were present in all 3 countries, some at levels considered potentially harmful (NDCI ≥ 0.1) (Figure 4; Tables 1 & 2), although no severe algal blooms (NDCI > 0.5) were detected (Tables 1 & 2; Appendix S2). Zambian lakes exhibited the highest NDCI values, reaching 0.34 (Figure 3c). The highest NDCI values for the large lakes in all 3 study areas were usually recorded along lake peripheries.

**TABLE 2:** Qualitative equivalent chlorophyll‐a concentrations associated with normalized difference chlorophyll index (NDCI) values (after Mishra & Mishra [2012, p. 405] and Binding et al. [2018])

NDCI range	Chlorophyll‐a range (mg/m^3^)	Interpretation
<–0.1	<7.5	Close to −1 optically clear
–0.1 to 0.0	7.5 to 16.0	Moderate algal biomass
0.0 to 0.1	16.0 to 25.0	Moderate to high algal biomass
0.1 to 0.2	25.0 to 33.0	Algal bloom risk
0.2 to 0.4	33.0 to 50.0	
0.4 to 0.5	>50.0	
0.5 to 1	Severe bloom	Severe algal bloom, with surface scum

### Lakes of high conservation priority

**FIGURE 5:**
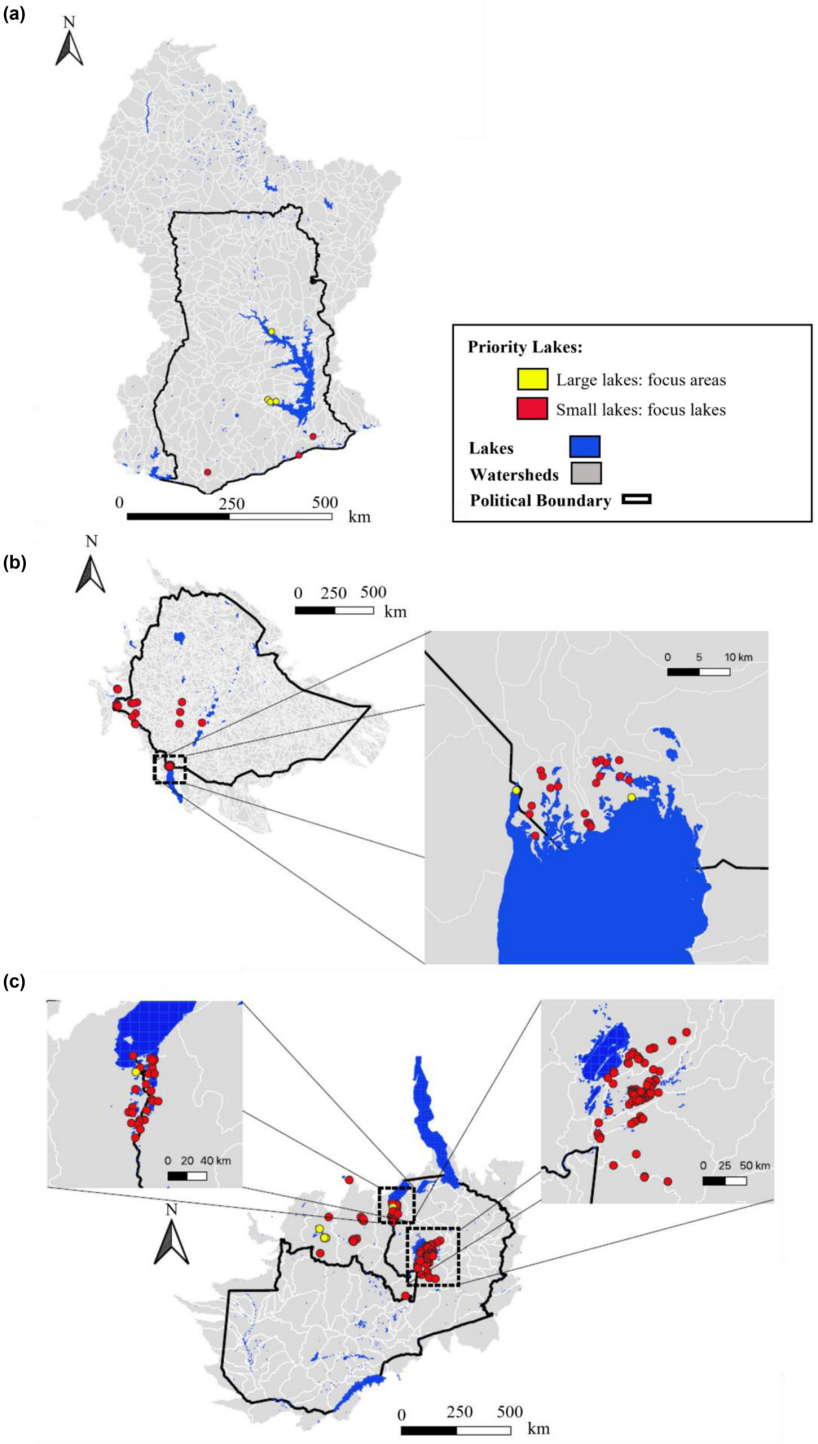
Lakes of conservation priority in (a) Ghana, (b) Ethiopia, and (c) Zambia, prioritized according to freshwater species richness and normalized difference chlorophyll index (NDCI) values. Many of the lakes identified in Ethiopia and Zambia are in watersheds that cross political boundaries.

Lakes identified as conservation priorities (i.e., median NDCI > 0.1 and freshwater species richness greater than the median value for the country) were found in all 3 countries. Of these, most were in Zambia; 129 small lakes (76.3% of all small lakes) and five 100‐km^2^ areas within larger lakes were identified as priorities. Ghana had the fewest priority lakes (3 small lakes and four 100‐km^2^ areas in larger lakes). In Ethiopia and Zambia, many of the priority areas straddled political boundaries. For instance, in Zambia a cluster of priority areas centered on the border with the Democratic Republic of the Congo (Figure 5c). In Ethiopia priority areas spanned parts of Kenya and Sudan (Figure 5b). The overlap between protected areas and priority lakes is shown in Appendix S4.

## DISCUSSION

Freshwater biodiversity currently faces numerous threats, most of which are expected to worsen under projected agricultural expansion and climate change in sub‐Saharan Africa (e.g., Odada et al., 2009; Sayer, Máiz‐Tomé, et al., 2018; van Soesbergen et al., 2019). Despite this, to our knowledge, research has not yet been conducted to prioritize freshwater systems for conservation in Africa according to species richness and threat (e.g., from eutrophication and HABs). In Ghana, Ethiopia, and Zambia (Figure 1), we identified 169 priority areas for conservation, which may involve threat *mitigation* in cases where degradation is yet to occur, or *restoration* if the habitat is already degraded, based on high levels of biodiversity exposed to potential HABs.

We went beyond previous work on climatic and vegetative patterns in freshwater fish biodiversity (Collen et al., 2014; Darwall et al., 2011) by identifying where high levels of freshwater biodiversity are spatially coincident with potential HABs (Figure 5). By incorporating freshwater‐dependent vertebrate species into our assessment (Figure 2), we also captured a wider range of taxa than has been investigated previously in biodiversity assessments in sub‐Saharan Africa (Holland et al., 2012; Máiz‐Tomé et al., 2017; McManamay et al., 2018). This is important because, in addition to the cascading effects of HABs on surrounding terrestrial ecosystems, algal bloom events can have direct and indirect effects on already‐vulnerable species highly dependent on freshwater ecosystems (Figures 3 & S1.3; Burkholder et al., 2018). Of course, obligate freshwater species will be at particularly high risk of the direct effects of algal blooms (e.g., oxygen depletion) and HABs (e.g., exposure to toxins).

Given the relatively recent and rapid expansion of agriculture in our focal countries, one might expect our observations to predate the strongest impacts of agriculture on freshwater ecosystems, highlighting the urgent need to prioritize lakes for the conservation of freshwater biodiversity. Our approach can be used to assess the coincidence between algal blooms and large numbers of threated species, thus providing a means through which to prioritize future conservation efforts for freshwater biodiversity (e.g., similar to terrestrial conflict hotspots identified by Molotoks et al. [2017]), particularly where funds for conservation are limited and cost‐effective methods are therefore necessary.

Based on our results, we suggest that management of water resources, including freshwater biodiversity, should be conducted at the watershed scale and may need to cross political boundaries. In Ethiopia and Zambia, for instance, many of the priority lakes were in transboundary watersheds (Figure 5), which suggests that collaboration and cooperation between countries and stakeholders will be imperative if the future effects of eutrophication and HABs are to be mitigated (Pittock et al., 2006). This may be best achieved through a well‐organized and structured participatory approach that integrates the values of key stakeholders, political leaders, and local communities in a unified water resource management plan (Pittock et al., 2006). Agreement among key stakeholders and countries on a shared vision for the conservation of these priority lakes would be especially important when the success of freshwater biodiversity conservation objectives, which align with United Nations Sustainable Development Goals, depends on associated work toward poverty alleviation and economic development (Bourne et al., 2016; Pittock et al., 2006; UN General Assembly, 2015). Furthermore, the use of an integrated approach and the production of a water resource management plan would factor in the socioeconomic and political restrictions of each country, as well as regions within countries, to support freshwater conservation initiatives by managing water resources at multiple geopolitical levels (Bourne et al., 2016; Pittock et al., 2006). The relationship between HABs and agricultural land use may also differ according to the region of study, which would be an interesting avenue for future work.

There are a number of limitations to our study. First, with our use of IUCN range maps to estimate the number of species per watershed, as in other broad‐scale biodiversity assessments, we assumed that a given species is present across its entire range. Thus, our measures of species richness may not reflect the actual number of species in local habitats (Herkt et al., 2017). Nevertheless, the IUCN dataset is the most regularly updated dataset used for large‐scale freshwater biodiversity assessments (Turak et al., 2017), and it captures broad‐scale biodiversity patterns. Second, the algal bloom index we used (NDCI) was an estimate of chlorophyll‐a. Thus, estimates of algal presence may be confounded by the presence of submerged aquatic plants and diatoms, especially in shallow areas, such as the edges of large lakes. A degree of caution is also required when assuming that chlorophyll‐a equates to algal biomass because the strength of this relationship varies (Felip & Catalan, 2000). However, chlorophyll‐a measurements are some of the most important indicators of water quality in lakes and waterbodies, and are used to estimate algal biomass with remote‐sensing methods, given the close relationship between chlorophyll‐a and eutrophication (Carvalho et al., 2013). The NDCI values derived for lake features were produced using top‐of‐atmosphere reflectance values, and no atmospheric corrections were applied, which might have influenced NDCI values for lakes. Nevertheless, early indications based on Sentinel‐2 data suggest that the top‐of‐atmosphere reflectance values in inland waters are suitable and perhaps the best metric for estimating water quality parameters, particularly chlorophyll (Toming et al., 2016).

Overall, we demonstrated the potential of remote‐sensing tools for use in prioritizing freshwater systems for conservation, given the coincidence of relatively severe algal blooms with potential hotspots of freshwater biodiversity in all 3 of the countries studied. In the absence of fine‐scale knowledge about where species occur in the lakes, our results could only highlight the potential for harmful effects on freshwater biodiversity at the national scale. Our results could thus be used to identify those areas that should be a priority for on‐the‐ground, field‐based research (e.g., in the south of Ghana, near Accra, western Ethiopia, and northeastern Zambia [Figure 5]), particularly if the lakes are not protected under current protected areas (Appendix S4) that may have been selected based on terrestrial biodiversity. In the past, work such as ours would have been hindered by a lack of satellite data with sufficient spectral, spatial, and radiometric resolution (Beck et al., 2016; Toming et al., 2016). These problems have been further complicated by the numerous reflectance algorithms developed to detect chlorophyll‐a pigments in water bodies, each with different advantages and limitations (Beck et al., 2016). The development of the NDCI (Mishra & Mishra, 2012), which can now be calculated with freely available remotely sensed data, enables one to investigate algal bloom prevalence in lakes across the globe. In doing so, we determined spatially explicit threats to freshwater biodiversity, likely posed by agricultural development in Ghana, Ethiopia, and Zambia, which is expected to expand in all 3 countries. We recommend these threats and the priority regions associated with them (Figure 5) be treated as urgent concerns for further investigation, given the spatial coincidence between algal bloom prevalence and higher numbers of threatened species in each of these countries (Figure 3).

## Supporting information

Appendix S0: Focal country locations
Figure S0.1: Map highlighting the locations of each of the focal countries in our study. This map was produced using ArcMap software (ESRI); here, the source data are from Global Administrative Areas (2012).
Appendix S1: Separating results for obligate freshwater species and freshwater‐dependent vertebrate species
Figure S1.1: The total number of freshwater‐dependent vertebrate species per watershed (associated with a – Ghana, b – Ethiopia, c – Zambia), estimated using IUCN species range information (IUCN, 2019). Plotted using QGIS (QGIS Development Team, 2019).
Figure S1.2: The total number of obligate freshwater species per watershed (associated with a – Ghana, b – Ethiopia, c – Zambia), according to IUCN freshwater HydroBASIN data (Lehner & Grill, 2013). Groups included in these counts are: fish, crayfish, crabs, molluscs, odonates, and shrimp. Plotted using QGIS (QGIS Development Team, 2019).
Figure S1.3: The total number of threatened species (Vulnerable, Endangered, and Critically Endangered; obligate freshwater species and freshwater‐dependent vertebrates) per watershed (associated with a – Ghana, b – Ethiopia, c – Zambia). Map production and plotting is as for Figure 2.
Appendix S2: Maximum NDCI values per study area
Figure S2.1: Maximum Normalized Difference Chlorophyll Index (NDCI) value of each lake in each of the study areas: a) Ghana, b) Ethiopia, and c) Zambia, and associated bordering countries. Algal blooms are deemed ‘severe’ when NDCI is greater than 0.5 but algal biomass is considered to be moderate to high in the range between −0.3 and 1 (Mishra & Mishra, 2012).
Appendix S3: Negative relationship between lake area and Normalized Difference Chlorophyll Index (NDCI)
We constructed linear models to analyse the relationships between different lake characteristics and NDCI values. Lake characteristics were obtained from the HydroLakes database (Messager et al., 2016). We found that lake area explained the most variation in NDCI values across all lakes (adjusted R2 = 0.31). After lake area, lake depth explained the most variation (adjusted R2 = 0.26) followed by shoreline ruggedness (adjusted R2 = 0.11). All explanatory variables were log10‐transformed for these analyses. Other candidate variables (e.g., shoreline length and total volume) showed strong collinearity with lake area, and so were not considered further.
Table S3.1: Results of three univariate models, where lake characteristics were used to explain the Normalized Difference Chlorophyll Index (NDCI) values of lakes within the study areas.
Appendix S4: Priority lakes in relation to Protected Area coverage
Figure S4.1: Distribution of priority lakes shown in Figure 5 in relation to Protected Areas in a) Ghana, b) Ethiopia, and c) Zambia, as included in the World Database on Protected Areas (WDPA, 2014) and therefore including classifications including, but not limited to: controlled hunting areas, National Forest Priority Areas, National Parks, Sanctuaries, and Wildlife Reserves.
