# Supplementary material for: Prioritizing conservation in sub‐Saharan African lakes based on freshwater biodiversity and algal bloom metrics
Source: Conserv Biol. 2022 May 26;36(5):e13914. doi: 10.1111/cobi.13914 (PMC9796571; doi:10.1111/cobi.13914)
Supplement: Supplementary file 1 — Appendix S0: Focal country locations Figure S0.1: Map highlighting the locations of each of the focal countries in our study. This map was produced using ArcMap software (ESRI); here, the source data are from Global Administrative Areas (2012). Appendix S1: Separating results for obligate freshwater species and freshwater‐dependent vertebrate species Figure S1.1: The total number of freshwater‐dependent vertebrate species per watershed (associated with a – Ghana, b – Ethiopia, c – Zambia), estimated using IUCN species range information (IUCN, 2019). Plotted using QGIS (QGIS Development Team, 2019). Figure S1.2: The total number of obligate freshwater species per watershed (associated with a – Ghana, b – Ethiopia, c – Zambia), according to IUCN freshwater HydroBASIN data (Lehner & Grill, 2013). Groups included in these counts are: fish, crayfish, crabs, molluscs, odonates, and shrimp. Plotted using QGIS (QGIS Development Team, 2019). Figure S1.3: The total number of threatened species (Vulnerable, Endangered, and Critically Endangered; obligate freshwater species and freshwater‐dependent vertebrates) per watershed (associated with a – Ghana, b – Ethiopia, c – Zambia). Map production and plotting is as for Figure 2. Appendix S2: Maximum NDCI values per study area Figure S2.1: Maximum Normalized Difference Chlorophyll Index (NDCI) value of each lake in each of the study areas: a) Ghana, b) Ethiopia, and c) Zambia, and associated bordering countries. Algal blooms are deemed ‘severe’ when NDCI is greater than 0.5 but algal biomass is considered to be moderate to high in the range between −0.3 and 1 (Mishra & Mishra, 2012). Appendix S3: Negative relationship between lake area and Normalized Difference Chlorophyll Index (NDCI) We constructed linear models to analyse the relationships between different lake characteristics and NDCI values. Lake characteristics were obtained from the HydroLakes database (Messager et al., 2016). We found that lake area explained the most vari [file COBI-36-0-s001.docx]

**Appendix S0: Focal country locations**

**
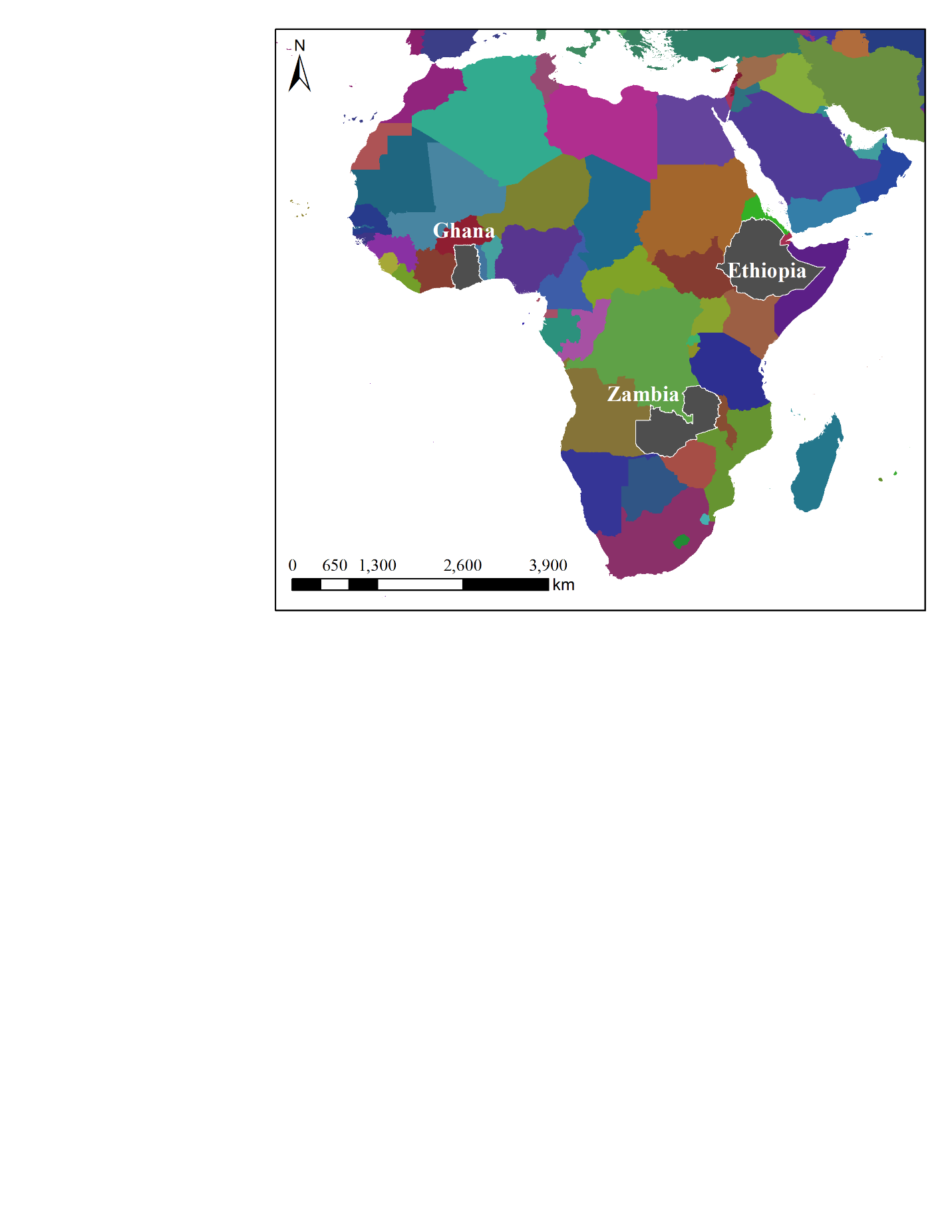
**

**Figure S0.1:** Map highlighting the locations of each of the focal countries in our study. This map was produced using ArcMap software (ESRI); here, the source data are from Global Administrative Areas (2012).

**Appendix S1: Separating results for obligate freshwater species and freshwater-dependent vertebrate species**

**
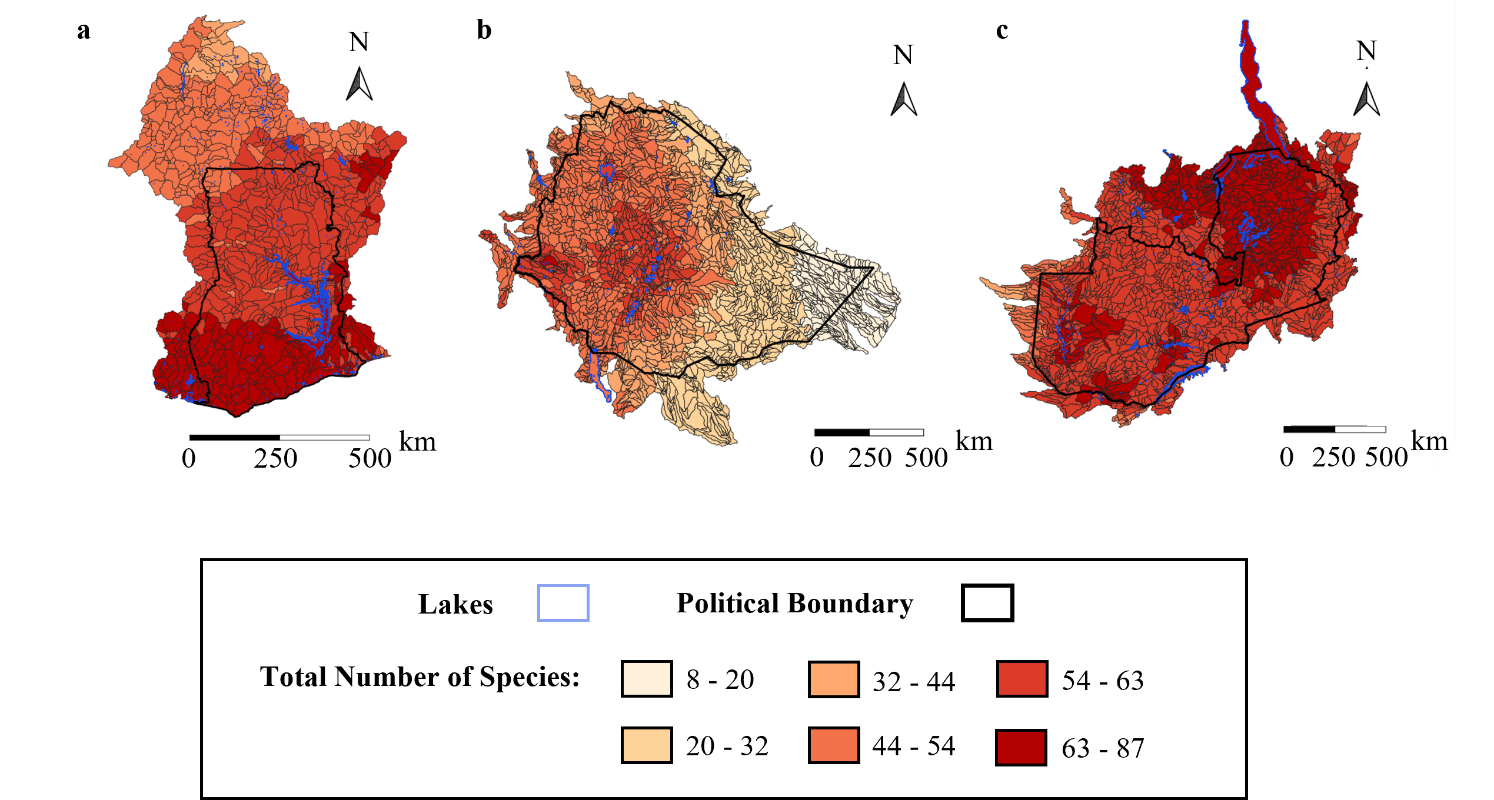
**

**Figure S1.1:** The total number of freshwater-dependent vertebrate species per watershed (associated with **a** – Ghana, **b** – Ethiopia, **c** – Zambia), estimated using IUCN species range information (IUCN, 2019). Plotted using QGIS (QGIS Development Team, 2019).

**
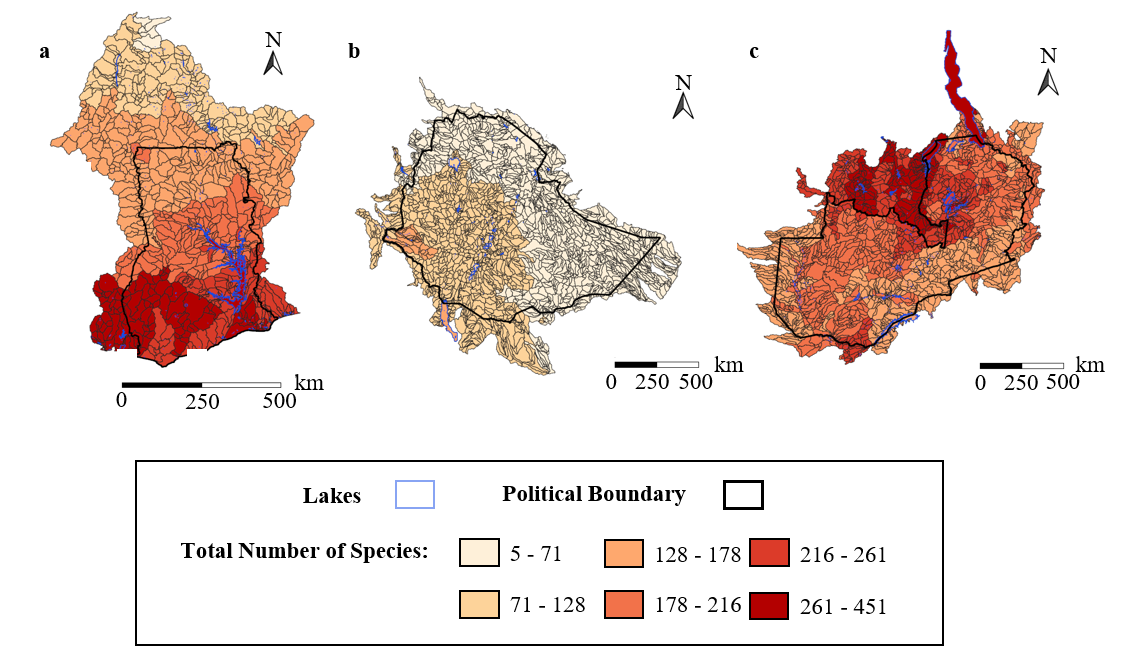
Figure S1.2:** The total number of obligate freshwater species per watershed (associated with **a** – Ghana, **b** – Ethiopia, **c** – Zambia), according to IUCN freshwater HydroBASIN data (Lehner and Grill, 2013). Groups included in these counts are: fish, crayfish, crabs, molluscs, odonates, and shrimp. Plotted using QGIS (QGIS Development Team, 2019).


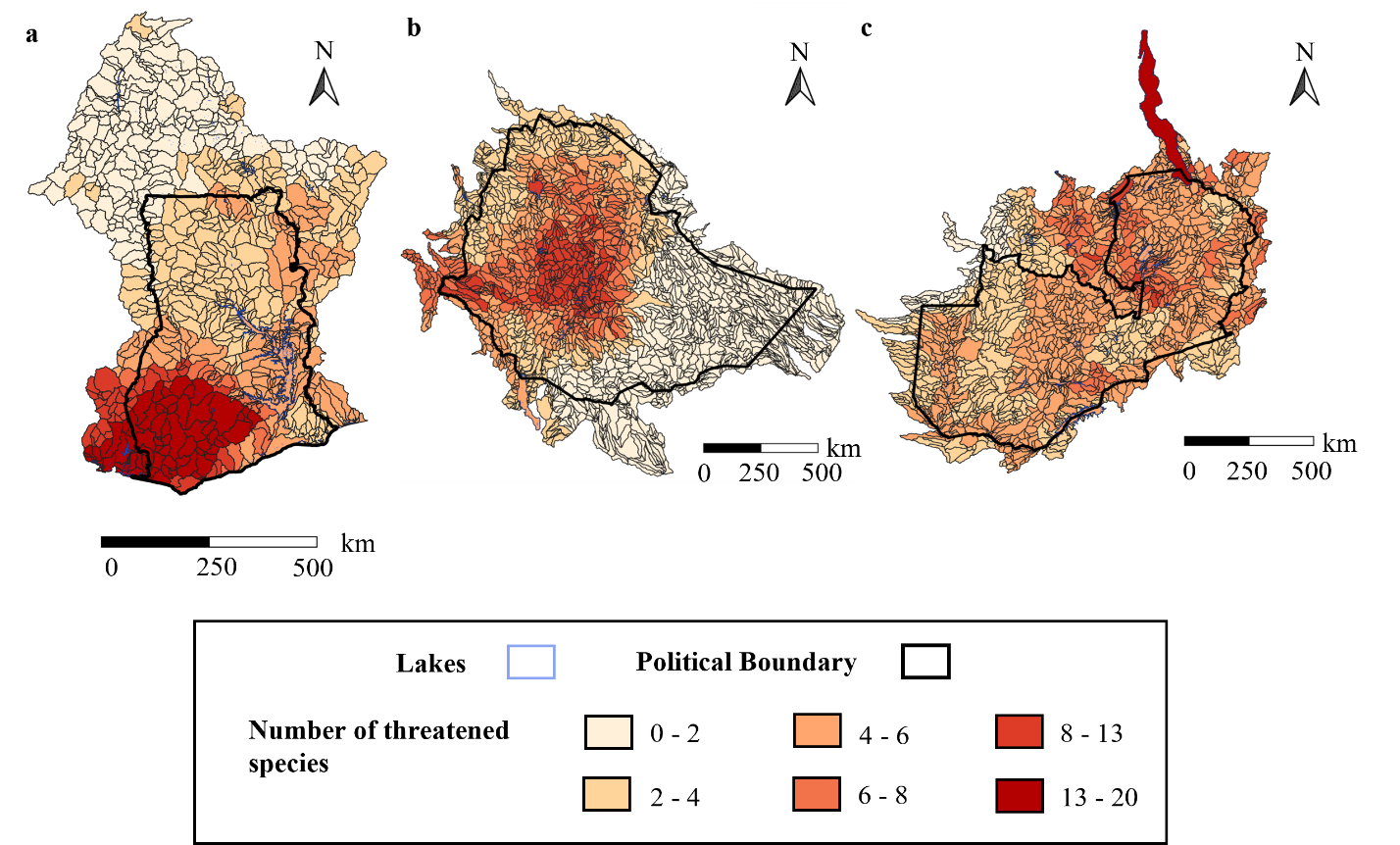


**Figure S1.3:** The total number of threatened species (Vulnerable, Endangered, and Critically Endangered; obligate freshwater species and freshwater-dependent vertebrates) per watershed (associated with **a** – Ghana, **b** – Ethiopia, **c** – Zambia). Map production and plotting is as for Figure 2.

**
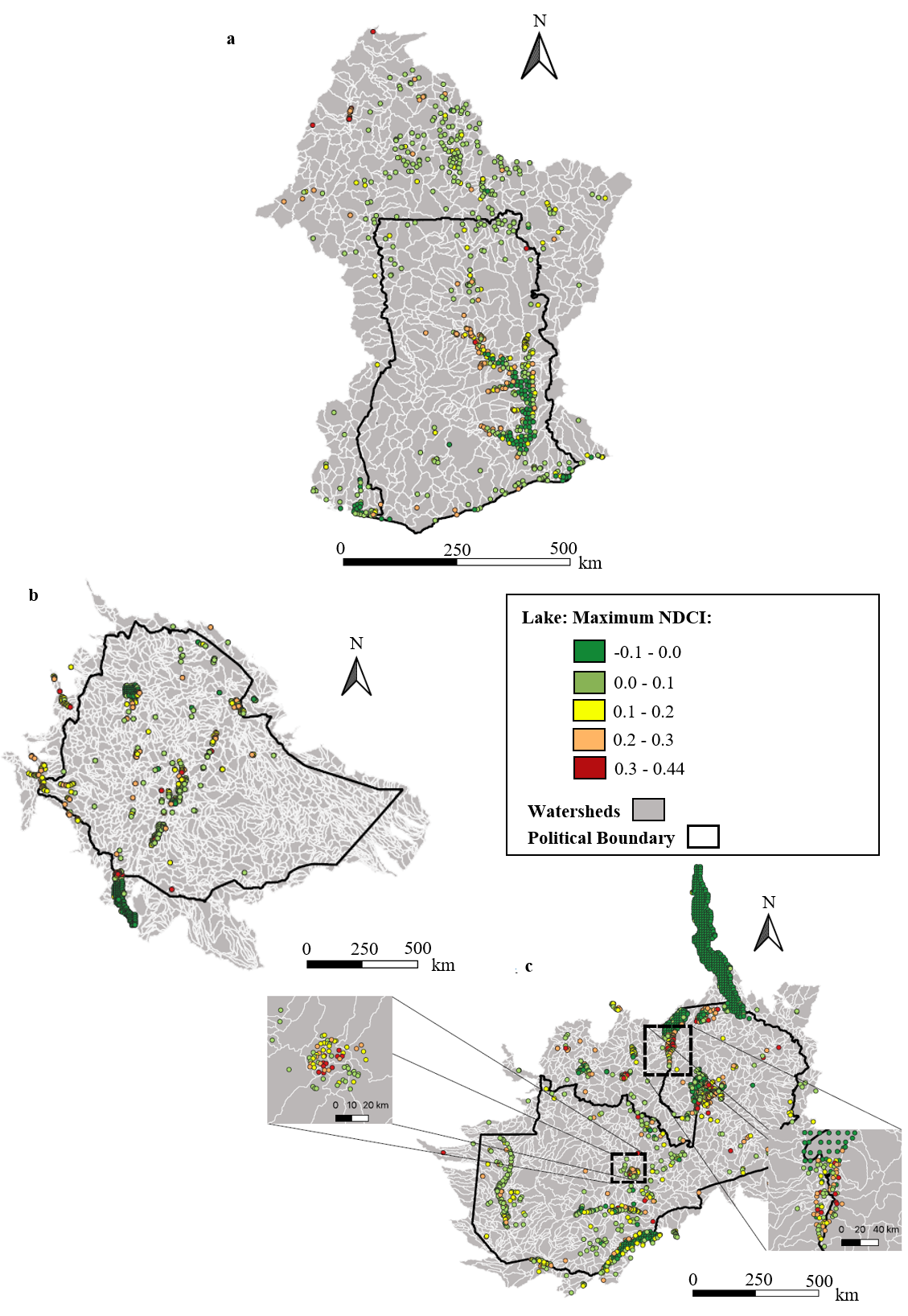
Appendix S2: Maximum NDCI values per study area**

**Figure S2.1:** Maximum Normalized Difference Chlorophyll Index (NDCI) value of each lake in each of the study areas: **a)** Ghana, **b)** Ethiopia, and **c)** Zambia, and associated bordering countries. Algal blooms are deemed ‘severe’ when NDCI is greater than 0.5 but algal biomass is considered to be moderate to high in the range between -0.3 and 1 (Mishra and Mishra, 2012).

**Appendix S3: Negative relationship between lake area and Normalized Difference Chlorophyll Index (NDCI)**

We constructed linear models to analyse the relationships between different lake characteristics and NDCI values. Lake characteristics were obtained from the HydroLakes database (Messager et al., 2016). We found that lake area explained the most variation in NDCI values across all lakes (adjusted R^2^ = 0.31). After lake area, lake depth explained the most variation (adjusted R^2^ = 0.26) followed by shoreline ruggedness (adjusted R^2^ = 0.11). All explanatory variables were log_10_-transformed for these analyses. Other candidate variables (e.g., shoreline length and total volume) showed strong collinearity with lake area, and so were not considered further.

**Table S3.1:** Results of three univariate models, where lake characteristics were used to explain the Normalized Difference Chlorophyll Index (NDCI) values of lakes within the study areas.

| **Linear Model** | **R^2^** | **Coefficient** | **Std. Error** | **t value** | **P** |
| --- | --- | --- | --- | --- | --- |
| NDCI.Average ~ log_10_ *(Lake Area)* | 0.31 | -0.019 | 0.001 | -36.84 | <0.001 |
| NDCI.Average ~ log_10_ *(Lake Depth)* | 0.27 | -0.04 | 0.001 | -32.72 | <0.001 |
| NDCI.Average ~ log_10_ (*Shoreline Ruggedness)* | 0.11 | -0.07 | 0.004 | -19.15 | <0.001 |

**Appendix S4: Priority lakes in relation to Protected Area coverage**


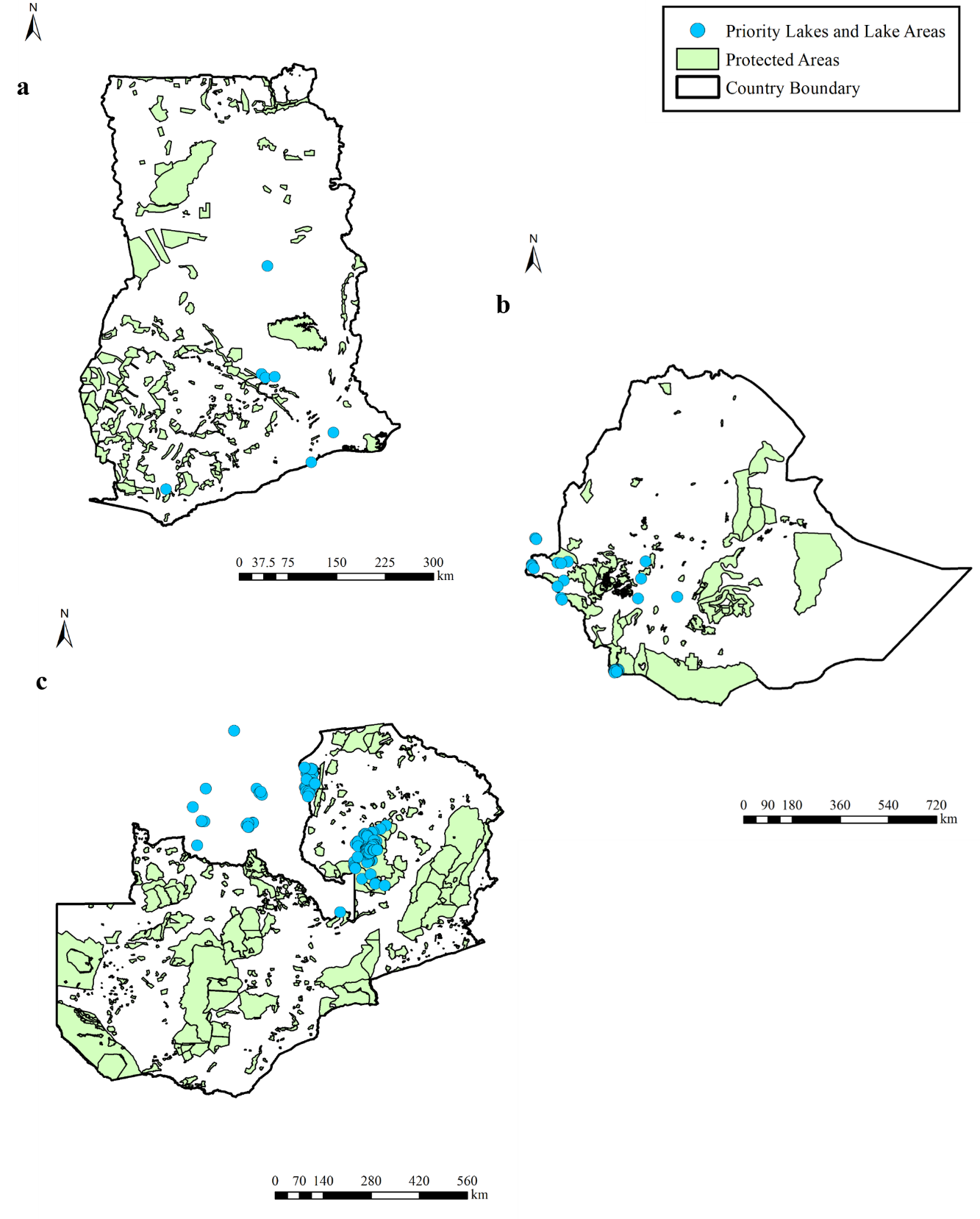


**Figure S4.1:** Distribution of priority lakes shown in **Figure** **5** in relation to Protected Areas in **a)** Ghana, **b)** Ethiopia, and **c)** Zambia, as included in the World Database on Protected Areas (WDPA, 2014) and therefore including classifications including, but not limited to: controlled hunting areas, National Forest Priority Areas, National Parks, Sanctuaries, and Wildlife Reserves.
